# Early Stage Preclinical Formulation Strategies to Alter the Pharmacokinetic Profile of Two Small Molecule Therapeutics

**DOI:** 10.3390/ph17020179

**Published:** 2024-01-30

**Authors:** Le An, Tom De Bruyn, Jodie Pang, Savita Ubhayakar, Laurent Salphati, Xing Zhang, Liling Liu, Ruina Li, Bryan Chan, Anwesha Dey, Elizabeth S. Levy

**Affiliations:** 1 Small Molecules Pharmaceutics, Genentech, 1 DNA Way, South San Francisco, CA 94080, USA; an.le@gene.com; 2 Drug Metabolism and Pharmacokinetics, Genentech, 1 DNA Way, South San Francisco, CA 94080, USA; de-bruyn.tom@gene.com (T.D.B.); pang.jie@gene.com (J.P.); ubhayakar.savita@gene.com (S.U.); salphati.laurent@gene.com (L.S.); zhang.xing@gene.com (X.Z.); liu.liling@gene.com (L.L.); li.ruina@gene.com (R.L.); 3 Discovery Chemistry, Genentech, 1 DNA Way, South San Francisco, CA 94080, USA; chan.bryan@gene.com; 4 Discovery Oncology, Genentech, 1 DNA Way, South San Francisco, CA 94080, USA; dey.anwesha@gene.com

**Keywords:** drug delivery, PO, IV infusion, IP, SC, small molecule, PK profile, exposure

## Abstract

Early stage chemical development presents numerous challenges, and achieving a functional balance is a major hurdle, with many early compounds not meeting the clinical requirements for advancement benchmarks due to issues like poor oral bioavailability. There is a need to develop strategies for achieving the desired systemic concentration for these compounds. This will enable further evaluation of the biological response upon a compound–target interaction, providing deeper insight into the postulated biological pathways. Our study elucidates alternative drug delivery paradigms by comparing formulation strategies across oral (PO), intraperitoneal (IP), subcutaneous (SC), and intravenous (IV) routes. While each modality boasts its own set of merits and constraints, it is the drug’s formulation that crucially influences its pharmacokinetic (PK) trajectory and the maintenance of its therapeutic levels. Our examination of model compounds G7883 and G6893 highlighted their distinct physio-chemical attributes. By harnessing varied formulation methods, we sought to fine-tune their PK profiles. PK studies showcased G7883′s extended half-life using an SC oil formulation, resulting in a 4.5-fold and 2.5-fold enhancement compared with the IP and PO routes, respectively. In contrast, with G6893, we achieved a prolonged systemic coverage time above the desired target concentration through a different approach using an IV infusion pump. These outcomes underscore the need for tailored formulation strategies, which are dictated by the compound’s innate properties, to reach the optimal in vivo systemic concentrations. Prioritizing formulation and delivery optimization early on is pivotal for effective systemic uptake, thereby facilitating a deeper understanding of biological pathways and expediting the overall clinical drug development timeline.

## 1. Introduction

The need for novel and effective therapies to combat cancer has led to the discovery of promising compounds targeting specific oncogenic pathways. However, translating these breakthroughs from preclinical research into clinical success has been impeded by significant challenges including poor pharmacokinetic (PK) profiles. Achieving the optimal balance between target exposure and minimal toxicity is essential to harness the full therapeutic potential of these challenging anti-cancer agents. Many promising anti-cancer agents have exhibited suboptimal PK properties, such as limited bioavailability (F), rapid clearance, and poor tissue penetration, thereby compromising their therapeutic efficacy and increasing the risk of adverse effects. In early stage molecule development, not only might the properties be less than ideal but the quantities of the compounds produced are also typically limited. Addressing these PK-related issues through molecule-specific formulation strategies is essential for the successful translation of these compounds into effective cancer therapies. 

In recent years, the pharmaceutical industry has observed an exponential increase in the discovery of novel therapeutic compounds targeting challenging disease pathways. Among these, the inhibition of transcriptional enhanced associate domain (TEAD) and hematopoietic progenitor kinase 1 (HPK1) has emerged as a promising approach to addressing various devastating diseases including cancer. 

The TEAD/YAP (yes-associated protein) pathway is an essential pathway for the development of oncology medicine because it plays a critical role in regulating cell proliferation, differentiation, and apoptosis [1]. The pathway is activated by the YAP transcriptional co-activator, which interacts with TEAD transcription factors to drive the expression of genes involved in cell growth and survival [1,2]. Aberrant activation of the TEAD/YAP pathway is a hallmark of many types of cancer, including liver, lung, breast, and ovarian cancers, and is often associated with poor prognosis and drug resistance [3]. Several drugs targeting the TEAD/YAP pathway are currently under development or in clinical trials, including small-molecule inhibitors of YAP–TEAD interaction, antibodies targeting the YAP or TEAD, and compounds that disrupt the upstream regulators of the pathway, such as the Hippo signaling pathway [4]. These drugs hold promise for the treatment of various types of cancer and may represent a new frontier in the development of oncology medicine.

The HPK1 biopathway is a signaling pathway involved in the regulation of immune response and cell proliferation [5,6]. It is activated by various stimuli, including cytokines, growth factors, and stress signals. Once activated, HPK1 initiates a cascade of intracellular events that lead to the activation of downstream effector molecules, such as transcription factors, kinases, and phosphatases. The HPK1 biopathway plays a crucial role in the regulation of T-cell activation and differentiation, as well as the activation of other immune cells such as B cells [7]. The HPK1 biopathway has been identified as a promising target for drug development aimed at treating various types of cancers, such as melanoma, lung, breast, and ovarian cancers, and it presents a viable option for developing cancer immunotherapy treatment [7,8,9]. Formulation development plays a critical role in the successful delivery of these drugs to the target site in the body. 

To address the challenge of achieving sufficient exposure while minimizing limitations such as toxicity, several strategies can be considered across different routes of administration and formulation methodologies. Routes that include intraperitoneal (IP), subcutaneous (SC), and intravenous (IV) serve as viable avenues to augment exposure in contrast to oral delivery. In the preliminary research phases, examining diverse administration routes can be instrumental in securing ample systemic exposure, facilitating the in-depth exploration of the biological functionalities of target molecules, and consequently refining these molecules for better efficacy. 

Unlike oral delivery, which requires the compound to navigate the complex terrain of the stomach and intestine in order to be absorbed into systemic circulation, the IP route provides a more direct absorption pathway [10]. Consequently, the IP route notably leads to quicker and increased absorption. Similarly, IV administration tends to yield amplified exposure and a peak plasma concentration (Cmax). In comparison, utilizing an IV infusion pump sidesteps first-pass metabolism in both the liver and gastrointestinal tract, directly introducing the drug into the bloodstream in a regimented manner. An example of an infusion pump is an iPRECIO pump, which not only confers granular dosing accuracy but also furnishes flexibility to extend drug delivery over prolonged durations, thereby setting the stage for a sustained-release profile.

By circumventing first-pass metabolism, SC administration has the potential to elevate the exposure of active pharmaceutical ingredients (APIs) that might otherwise be compromised in the intestinal environment or be subjected to metabolism. Moreover, sophisticated formulation strategies can be leveraged to fine-tune SC delivery, aiming for a more uniform and predictable drug concentration in the bloodstream over time, which is facilitated through the gradual drug release from the subcutaneous reservoir. One approach is to develop extended-release formulations for SC administration, which can maintain the desired exposure for the duration of the study while reducing adverse effects. 

Here, we explored formulation strategies for both IV infusion administration through the iPRECIO pump and SC administration. Both of these parenteral routes offer distinct advantages over conventional oral administration, such as the rapid onset of action, increased bioavailability, and reduced first-pass metabolism, which could significantly alter the PK profiles of the compounds.

In vivo studies were conducted using two distinct compounds to assess the PK profiles of these formulation strategies and routes. The results of these studies will provide valuable insights into the PK profiles of G7883 and G6893 by comparing PO, IP, IV, and SC routes with different proposed formulation strategies, thus aiming to achieve the proof of concept in PKPD and efficacy studies. Furthermore, the work here will guide strategic choices based on the compound’s characteristics, aiding in determining the optimal delivery method and formulation strategy.

In this paper, we present a focused investigation into the formulation strategies employed to enhance the PK profiles of two distinct anti-cancer agents as follows: G7883, which is targeted for TEAD, and G6893, which is targeted for HPK1, each targeting unique signaling pathways. These compounds hold tremendous promise in treating specific types of cancer, but their preclinical development has been hindered by their distinct PK challenges. G7883, for instance, has exhibited poor oral bioavailability due to low aqueous solubility and extensive first-pass metabolism. G6893, despite adequate bioavailability, shows moderate systemic clearance, thereby resulting in insufficient exposure to the target tissue. The successful enhancement of the PK profiles of G7883 and G6893 are expected to advance the therapeutic potential of the targets to contribute to generating improved therapeutics. By tailoring formulation strategies to the unique challenges presented by these compounds and exploring IV infusion and SC delivery routes, we seek to establish a foundation for more efficient drug development processes. 

## 2. Results and Discussion

During the initial stages of developing therapeutic molecules, various chemical compounds with diverse properties are synthesized. As the process advances, the objectives shift from that of primarily understanding the biological pathways using tool molecules to that of optimizing the chemical properties to create a clinical compound. This discussion concentrates on the development strategies of two distinct model compounds that share a common goal of utilizing different formulations and route strategies to modify and enhance the systemic PK profiles of these molecules. This, in turn, allows for a better comprehension of the biological pathway and facilitates the optimization of chemical matter with improved properties.

### 2.1. Physiochemical Property and Compound Characterization

**Figure 1:**
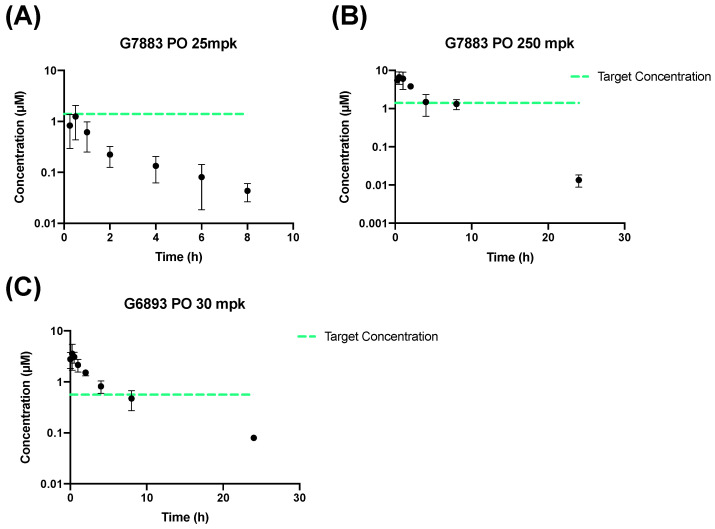
Mouse PK data administered through the PO route in the MCT vehicle. G7883 dosed at (**A**) 25 mpk and at (**B**) 250 mpk. (**C**) G6893 dosed at 30 mpk. Data represent the mean ± SD, N = 3. Target concentration = IC50.

G7883 is a small molecule chemically designed to bind and inhibit the interaction between TEAD and YAP/TAZ (transcriptional coactivator with PDZ-binding motif) with a MW of 500 g/mol and logP of 1.83 (Table 1, Figure S1) [11]. G7883 is a crystalline free base characterized by moderate permeability and a solubility that is 89 µg/mL in phosphate buffer saline (PBS). Although the compound has low oral bioavailability (Figure 1), the compound has high potency in cellular assays with a half-maximal inhibitory concentration (IC50) of 1.4 µM [12]. As a result, we investigated strategies to increase and alter the PK profile to achieve the desired IC50 coverage for greater than 10 h. 

We compared the G7883 molecule with another oncology compound targeted to inhibit the HPK1 signaling pathway, G6893. Similarly to G7883, G6893 is also a crystalline free base but has low solubility and high permeability (Table 1, Figure S1). G6893 is a potent molecule with an IC50 of 0.56 µM. Here, we compare the formulation development strategies to improve the systemic coverage (time above the IC50) for both compounds in preclinical species. 

### 2.2. PO and IP Administration

Both compounds were initially evaluated for bioavailability through oral delivery. The process of developing clinical compounds typically favors oral delivery due to its less invasive nature, ease of administration, and increased patient adherence [12,13]. With regard to preclinical studies, oral administration is favored because it closely mimics the clinically desired administration route and simplifies the administration and dosage process. To investigate the oral bioavailability and PK properties of G7883 and G6893, three groups of mice were dosed through oral gavage in a MCT vehicle. The MCT vehicle, which was composed of polymer and surfactant, was utilized to improve the suspendability of the compounds. To achieve the desired coverage based on covering the IC_50_ for G7883, the first condition tested was that of increasing the dose level from 25 mpk to 250 mpk dosing through the PO route (Figure 1A,B). At the higher dose, the exposure increased was approximately linear with improved coverage, and the concentration maintained was above the IC50 for 6 h. However, the bioavailability remained low (approximately 7%) with a plasma half-life of 2 h (Table 2). On the other hand, for G6893 at a 30 mpk dose, the F was moderate at 45.5%, thereby maintaining the IC50 for ~6 h (Figure 1C). Although oral administration is the preferred route, there are multiple barriers that can result in poor oral bioavailability, reducing systemic concentrations [14]. Compounds delivered orally must withstand the harsh environments in the gut and maintain sufficient solubility in the intestinal space to allow for absorption [12]. Beyond the challenges of solubility, inherent molecular characteristics, such as low permeability, can limit absorption. After these molecules are absorbed from the intestines, they face the liver where metabolism can occur, which is often referred to as first pass metabolism [14,15]. Investigating alternative routes of delivery with lower barriers to absorption into the systemic circulation, especially in the early stages of drug discovery, can lead to diverse PK profiles. This exploration aids in understanding the biological impact when the compound is tested in a preclinical setting. 

**Figure 2:**
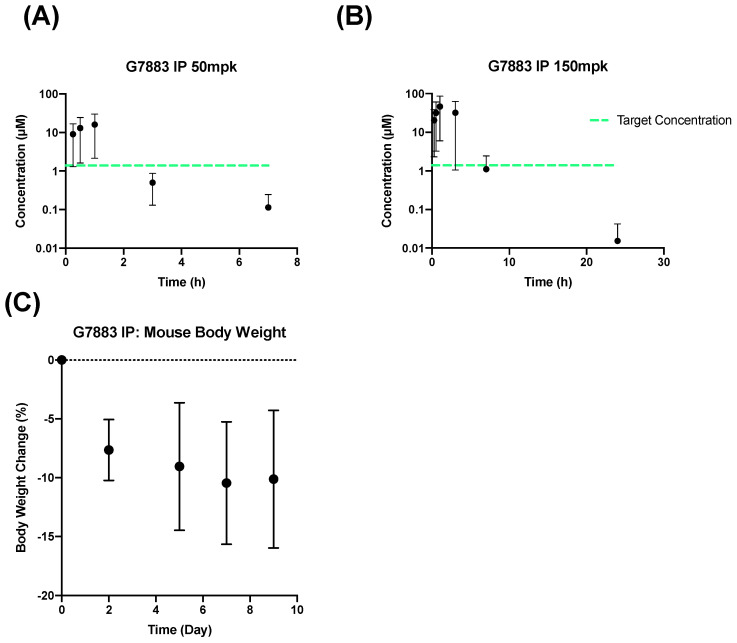
IP Administration of G7883 in a mouse at (**A**) 50 mpk and (**B**) 150 mpk as well as (**C**) body weight change from a multiday study dosed at 150 mpk BID. Data represent the mean ± SD, N = 3. Target concentration = IC50.

Considering the poor oral bioavailability of G7883, an alternative method of administration, that of intraperitoneal (IP) delivery, was chosen to bypass potential intestinal absorption challenges. Although the clinical translational value of IP administration in rodents is constrained by its limited utilization in clinical practices, it provides a rapid, uncomplicated procedure that is often adopted in initial proof-of-concept studies to assess target engagement [10]. The rate of absorption subsequent to IP administration surpasses both the SC and oral routes [16]. In a pursuit to explore non-oral routes that may enhance exposure, G7883 was administered in a MCT vehicle through the IP route to two separate groups of mice at a dose of 50 and 150 mpk (Figure 2A,B). Compared with oral dosing, the AUC coverage from IP administration was significantly increased by approximately 10-fold from a 250 mpk PO dose with an AUC of 25 h·µM to that of 217 h·µM with an IP dose of 150 mpk (Table 3). Although the AUC coverage increased, the time over the target IC50 remained the same with coverage up to 6 h for the 150 mpk IP group due to the rapid elimination and shorter half-life of 1.5 h. 

Additionally, in a study spanning over 10 days, significant decreases in the body weights of mice were observed, thereby constraining the maximal dosage that could be administered through the IP route (Figure 2C). This weight reduction is possibly attributable to the much higher Cmax reached following the IP administration of G7883 as compared with the PO administration. IP administration circumvents several absorption barriers, such as that of improved and faster absorption, due to the direct vascular access of the peritoneal space compared with the intestinal barrier. Consequently, we undertook investigations into alternate strategies to boost absorption and extend the time of the IC50 coverage.

### 2.3. IV Infusion Administration and Pharmacokinetics

Small molecules often fail to demonstrate the most advantageous pharmacokinetic (PK) profiles as many compounds are cleared rapidly from the body, thus diminishing their therapeutic efficacy. This is often a concern for molecules in early drug discovery stages [17]. Various formulation and delivery strategies have been devised to modify the systemic PK profiles of drug molecules, encompassing nanoparticles, microparticles, lipid and polymer conjugation, implanted infusion pump device technologies, and subcutaneous depots [17,18,19]. Here, we examined two strategies aimed at maintaining the drug concentration at a designed target concentration for the maximum duration potentially needed for proof of concept studies. The initial approach involved using an implantable device, the iPRECIO pump that allows for a controlled and sustained infusion, as opposed to a typical bolus IV injection [20]. The infusion pump strategies can be a promising tool to achieve the desired delivery profile of a compound, given the solubility requirements based on the intended target release and PK profile. The iPRECIO pump system requires formulation development as the reagent systems must be compatible with the device, such as strong acids and bases, solvents, complexing agents, and surfactants. The pump system typically requires a solution formulation at the predicted target concentration to cover the IC50. G7883 was administered with the iPRECIO pump over 176 h in mice at a concentration of 3.3 mg/mL as a solution in a 20% DMSO, 80% PEG400 vehicle. The iPRECIO pump system was also tested with G6893 at a concentration of 2.5 mg/mL as a solution in a 100% PEG400 vehicle, which was infused over 96 h.

**Figure 3:**
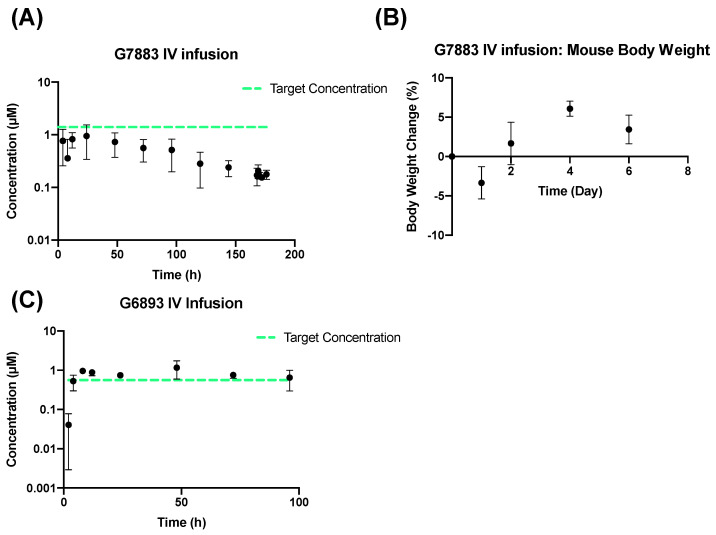
Mouse IV infusion iPRECIO pump. G7883 administered at 1.54 mg/h.kg in a 20% DMSO, 80% PEG400 (**A**) G7883 plasma concentration, (**B**) body weight data, and (**C**) G6893 dosed at a rate of 0.5 mg/kg/h in 100% PEG400. Data represent the mean ± SD, N = 3. Target concentration = IC50.

Although the iPRECIO pump substantially modified the PK curve for G7883, resulting in sustained delivery and prevention of body weight loss over time, the plasma concentrations remained below the target IC50 (Figure 3A,B). Due to G7883’s poor solubility, the ability to sufficiently solubilize it at a higher concentration while maintaining compatibility with the iPRECIO pump system reached its limits. In contrast, the iPRECIO pump successfully maintained the target concentration profile for G6893, partially due to the lower IC50 and, accordingly, the lower required systemic concentration to achieve the target coverage over time (Figure 3C). Based on the physicochemical properties of the compounds, G6893 proved more amenable for application with the iPRECIO pump system compared with G7883.

### 2.4. Subcutaneous Depot Development and Pharmacokinetics

**Figure 4:**
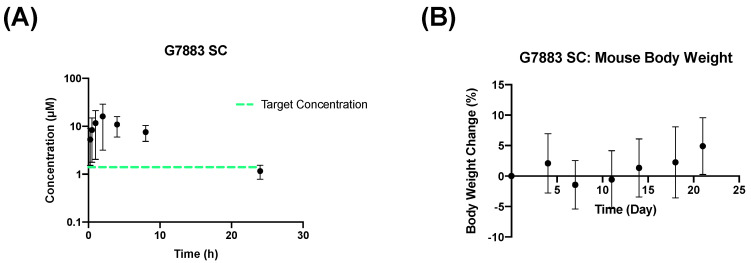
Mouse SC data of G7883 administered in sunflower oil at 250 mpk. (**A**) PK data and (**B**) body weight data. Data represent the mean +/− SD, N = 3. Target concentration = IC50.

During various developmental stages, subcutaneous administration could be an alternative strategy to achieve a prolonged release profile through parenteral delivery. Given that neither the PO nor IV administration approaches tested for G7883 yielded the desired exposure profile, we chose to explore the use of a sustained-release SC formulation based on a slow-eluting oil depot. Subcutaneous oil formulations have demonstrated their ability to function as depots, with absorption dictated by factors like solubility, diffusion, and entry into the bloodstream [21]. The release profile can be optimized by manipulating various parameters, such as particle size, surface properties, and the dosing dispersion medium [22]. The optimization of long-acting injectables for subcutaneous delivery confers several advantages over oral and intraperitoneal routes of delivery. Subcutaneous depots can function as a slow sustained-release system, resulting in less frequent dosing and simultaneously circumventing the first-pass metabolism associated with oral and intraperitoneal administration. Here, we developed a formulation of G7883 in sunflower oil that was dosed at 250 mpk through the SC route. In sunflower oil, G7883 was a suspension where a certain amount of the compound was dissolved in the oil while the remaining compound was in a solid form. Compared with the 250 mpk PO dose, the SC group had significantly higher bioavailability, achieving 46% (Figure 4A). Additionally, the AUC increased more than 5-fold to 137 h·µM when administered as a SC oil formulation compared with 25 h·µM orally (Table 4). The Cmax, although higher than the PO route, was suppressed compared with G7883 administered through the IP route. The Cmax for the 250 mpk SC group was reduced approximately 4-fold compared with the IP group that was dosed at 150 mpk.

G7883 dosed through a SC route was tolerated after administration for over 21 days (Figure 4B). Additionally, the SC group dosed at 250 mpk resulted in a significantly longer half-life compared with the IP one dosed at 150 mpk and the PO one dosed at 250 mpk, being approximately 4.5-fold and 2.5-fold longer, respectively. The combination of increased bioavailability and an extended half-life facilitated the IC50 coverage for nearly 24 h. This substantial enhancement in coverage, coupled with improved tolerability, enabled critical in vivo efficacy studies [11]. 

SC depots may serve as a beneficial approach during the initial development phases, providing a less invasive solution in comparison with the iPRECIO pump. Nonetheless, the scope of subcutaneous delivery is not without its limitations; the range of compounds that are viable for this method may be confined by their intrinsic properties. Furthermore, the release profile is dictated by the compounds’ physicochemical properties and corresponding formulations. Conversely, the iPRECIO pump consistently demonstrates its capacity to reliably obtain the intended release profile.

## 3. Materials and Methods

### 3.1. Materials

G7883 (5-(4-cyclohexylphenyl)-3-[3-(fluoromethyl)azetidine-1-carbonyl]-2-(3-methyl pyrazin-2-yl)-4H-pyrazolo[1,5-a]pyrimidin-7-one) and G6893 ([rac-(3R,4S)-4-methyltetra hydrofuran-3-yl]N-[8-amino-7-fluoro-6-(8-methyl-2,3-dihydro-1H-pyrido[2,3-b][1,4] oxazin-7-yl)-3-isoquinolyl]carbamate) compounds were synthesized at Genentech (South San Francisco, CA, USA). Polyethylene glycol 400 UPS grade, sunflower oil, corn oil, and polysorbate 80 NF grade were from Spectrum™ Chemical (New Brunswick, NJ, USA). Organic solvents (Dimethyl Sulfoxide and Ethanol), Methyl cellulose M6385 A15 LV, and (2-Hydroxypropyl)-β-cyclodextrin (HP-β-CD) were from Sigma-Aldrich (St. Louis, MO, USA). Micro-infusion pump iPRECIO SMP-300 was purchased from ALZET (Cupertino, CA, USA).

### 3.2. PO and IP Vehicle and Formulation Preparation

A total of 0.6%* w*/*v *Methylcellulose, 0.2%* w*/*v *Tween 80 in water (MCT) was prepared by slowly adding Methylcellulose (Methylocel A15 LV; CAS 9004-67-5, Part ID: 64605-500G-F 27.5-31.5% Methoxyl basis, Sigma-Aldrich, St. Louis, MO, USA) in small portions to prevent clumping while stirring into water at 70–80 °C. Once a homogeneous suspension formed, Tween 80 (Polysorbate 80; CAS#9005-65-6 (Polyoxyethylene (20) Sorbitan Monooleate; Tween 80) Part ID: PO138, Spectrum Chemical, New Brunswick, NJ, USA) was added and stirred until fully dissolved. Vehicle was filtered through 0.22 µm PES (Corning REF no. 431098) prior to adding to the compound for dosing. Respective compound was added based on the target concentration to the MCT vehicle and stirred at 35 °C until the power was well-dispersed. Formulations were homogenized through a polytron homogenizer and then sonicated in a water bath sonicator until a homogeneous suspension was achieved.

### 3.3. PO Study

The pharmacokinetics of G7883 were determined in female CD-1 mice obtained from Charles River Laboratories (Hollister, CA, USA) with a body weight ranging from 20 to 25 g. The pharmacokinetics of G6893 were determined in female C57BL-6 mice (Lingchang/Vital River Laboratory Animal Co., Ltd., Shanghai/Beijing, China) with a body weight ranging from 20 to 35 g. Three mice were given an oral dose of G7883 formulated in 0.6% Methylcellulose, 0.2% Tween80 in water (MCT) at 25 mg/kg and 250 mg/kg with a dosing volume of 10 mL/kg. Food and water were available ad libitum to the animals. Three mice were given an oral dose of G6893 formulated in 0.6% methylcellulose; 0.2% Tween80 in water with pH adjusted to 2.5 at 30 mg/kg and a dose volume of 5 mL/kg. For both compounds, G7883 and G6893, 15 µL of blood was collected serially at 0. 25, 0.5, 1, 2, 4, 6, 8, and 24 h post-dose and diluted in 60 µL of 1.7 mg/mL potassium K2EDTA water. The frozen blood samples were stored at −70 °C or lower until analysis.

### 3.4. IP Study

G7883 was dosed in three female NSG mice in-house with body weight ranging from 20 to 25 g. Three mice were given an IP dose of G7883 formulated in the MCT vehicle at 50 mpk and 150 mpk with a 5 mL/kg dosing volume. A total of 10 µL of blood was collected serially at 0.25, 0.5, 1, 3, 7, and 24 h post-dose and diluted in 40 µL of the potassium K2EDTA water. The frozen blood samples were stored at −70 °C or lower until analysis.

### 3.5. IV Infusion Study

Vehicle and Formulation Preparation

Based on the target concentration, G7883 was screened at 3.3 mg/mL in three different vehicles of 100%PEG400, 10%DMSO in 90%PEG400, and 20%DMSO in 80%PEG400 to generate a stable solution. G7883 achieved a solution at the target concentration of 3.3 mg/mL in 20%DMSO in 80%PEG400 and maintained that solubility when diluted in PBS buffer. A total of 50% DMSO is the maximum amount compatible with the iPRECIO pump system. Based on the target concentration, G6893 was screened at 5 mg/mL in four different vehicles as follows: 100% PEG400, 20% DMSO in 80% PEG400, 20% DMA and 40% PG in 40% PEG400, and 10% EtOH and 20% HPbCD in 70% water at pH 4.5. G6893 achieved a solution at the target concentration of 5 mg/mL in 100% PEG400 and maintained a solution without precipitation after dilution in PBS buffer.

Formulations selected for G7883 in the iPRECIO pump IV infusion study were 20% Dimethyl Sulfoxide (DMSO): 80% Polyethylene glycol 400 (PEG400) at 3.3 mg/mL. Five female C57BL-6 mice were dosed at 0.8 mg/kg/h with an infusion rate of 0.2 mL/h/kg (5 uL/h). The pump was refilled every 24 h with a study duration of 7 days. The formulation selected for G6893 in the iPRECIO pump IV infusion study was 100% PEG400 at pH 6 at 2.5 mg/mL. Three female C57BL-6 mice with a body weight ranging from 17 to 18 g were dosed at 0.5 mg/kg/h with an infusion rate of 0.2 mL/h/kg (5 µL/h). The pump was refilled every 24 h with a study duration of 5 days.

### 3.6. Subcutaneous (SC) Study

Vehicle and Formulation Preparation

G7883 was screened in different oil vehicles including corn, coconut, paraffin, soybean, and sunflower oil. The suspendability and solubility were assessed and sunflower oil was determined to be optimal one based on the superior suspendability and moderate solubility.

For subcutaneous administrations, G7883 was formulated in sunflower oil as a suspension and dosed at 250 mg/kg with a dosing volume of 5 mL/kg. Food and water were available ad libitum to animals. Blood samples were collected post-dose into tubes containing K2EDTA as an anticoagulant. Samples were taken from three different animals at each time point. After the blood was mixed with K2EDTA, the samples were stored at −80 °C until analysis.

### 3.7. Bioanalytical Methods

All plasma and cell media concentrations of G7883 and G6893 were determined using liquid chromatography–tandem mass spectrometry after protein precipitation with acetonitrile and injection of the supernatant onto the column (Waters ACQUITY UPLC BEH C18 1.7 μm, 2.1 × 50 mm for G7883 and G6893). The aqueous mobile phase was 0.1% formic acid, 2 mM ammonium formate in water/acetonitrile (*v:v*, 95:5), and the organic mobile phase was 0.1% formic acid, 2 mM ammonium formate in acetonitrile/water (*v:v*, 95:5). An AB Sciex API 4000 Triple Quad instrument was used for detecting G7883 and G6893 (Figure S2). The electrospray ionization source in both columns was operated in the positive ion and multiple reaction monitoring (MRM) detection modes. The MRM transitions for G7883 and G6893 were *m/z* 462.2 (Q1) and 253.10 (Q3) and m/z 454.2 (Q1) and 325.9 (Q3), respectively. The dynamic range of both assays was 0.50–50.0 ng/mL, and the total run time was 1.6 min. The lower limit of quantitation of the assay was 5.1 ng/mL, and the dilution quality control concentration was set at 10 ng/mL with acceptance criteria for accuracy and precision of 25%.

All plasma and cell media concentrations of G7883 were determined using liquid chromatography–tandem mass spectrometry after protein precipitation with acetonitrile and injection of the supernatant onto the column (Phenomenex Kinetex XB-C18, 30 × 2.1 mm, 2.6 µm). The aqueous mobile phase was 10 mM of ammonium formate in water, and the organic mobile phase was 0.1% formic acid in acetonitrile. An AB Sciex API 5500 Triple Quad instrument was used for detecting G7883. The electrospray ionization source in both columns was operated in the positive ion and MRM detection modes. The MRM transitions for G7883 were *m/z* 462.2 (Q1) and 253.10 (Q3), respectively. The dynamic range of the assay was 0.50–50.0 ng/mL, and the total run time was 2 min. The lower limit of quantitation of the assay was 3.05 ng/mL, and the dilution quality control concentration was set at 10 ng/mL with acceptance criteria for accuracy and precision of 25%.

## 4. Conclusions

In this study, we investigated multiple different formulation approaches for two anticancer therapeutic molecules with challenging oral pharmacokinetic characteristics. G7883’s rapid clearance and short half-life hindered its advancement to efficacy studies. However, by exploring various administration techniques and formulations, we were able to achieve an improved PK profile by dosing a sunflower oil vehicle through the SC route to create a depot. This work paved the way for further development to evaluate the efficacy of G7883, which was otherwise infeasible due to low exposure. Similarly, for G6893, another promising agent with limited solubility, we successfully applied an off-the-shelf iPRECIO pump drug delivery system to enable controlled systemic exposure through IV infusion. These results highlight the importance of investigating alternative delivery methods and strategies to enable critical in vivo studies to facilitate a deeper understanding of the biological pathways. This is especially true for nascent molecules in the early discovery phase. By evaluating more compounds with varied physicochemical attributes using these methods, we may be able to identify promising early stage strategies for preclinical advancement.

## Figures and Tables

**Table 1:** Physiochemical properties of G7883 and G6893.

	G7883	G6893
MW (g/mol)	500.57	450
Water Solubility (µg/mL)	85	<1
Log P	1.83	2.69
pKa	5.51, 4.83	2.53, 3.87
Permeability	5.79 × 10^−6^ cm/s	15 × 10^−6^ cm/s

**Table 2:** Non-compartmental analysis of G7883 (25 and 250 mpk) and G6893 (30 mpk) administered through PO.

Pharmacokinetic Parameter	PO 25 mpk (G7883) (Mean ± SD)	PO 250 mpk (G7883) (Mean ± SD)	PO 30 mpk (G6893) (Mean ± SD)
AUC_0 to 8_ (h·µM)	1.99 ± 0.40	25.1 ± 5.43	19.5
Cmax (µM)	1.25 ± 0.82	6.85 ± 2.24	3.80 ± 1.68
Tmax (h)	0.5	0.583 ± 0.382	0.667 ± 0.289
t_1/2_ (h)	2.17 ± 0.73	2.74 ± 0.201	3.07 ± 1.92
F (%)	6.18 ± 1.24	7.80 ± 1.69	45.5 ± 14.0

**Table 3:** Non-compartmental analysis of G7883 (50 and 150 mpk) administered through an IP route.

Pharmacokinetic Parameter	IP 50 mpk (Mean)	IP 150 mpk (Mean)
AUC_0 to t_ (h·µM)	31.2	217
Cmax (µM)	23.9	69.2
Tmax (h)	1	1
t_1/2_ (h)	0.895	1.53

**Table 4:** Non-compartmental analysis of G7883 (250 mpk) administered through a SC route.

Pharmacokinetic Parameter	SC 250 mpk (G7883) (Mean ± SD)
AUC_0 to t_ (h·µM)	137 ± 51.1
Cmax (µM)	16.7 ± 12
Tmax (h)	2.67 ± 1.15
t_1/2_ (h)	6.94 ± 2.93
F (%)	46.4 ± 13.2

## Data Availability

Data available upon request.

## Supplementary Materials

The following supporting information can be downloaded at: https://www.mdpi.com/article/10.3390/ph17020179/s1. Figure S1: Chemical structures of (a) 7883 and (b) G6893; Figure S2: Chromatograms of Blank (a) G7883 and (b) G6893, a standard (c) G7883 and (d) G6893, and a sample (e) G7883 and (f) G6893.
