# Supplementary material for: Early Stage Preclinical Formulation Strategies to Alter the Pharmacokinetic Profile of Two Small Molecule Therapeutics
Source: Pharmaceuticals (Basel). 2024 Jan 30;17(2):179. doi: 10.3390/ph17020179 (PMC10892288; doi:10.3390/ph17020179)
Supplement: Supplementary file 1 [file pharmaceuticals-17-00179-s001.zip › pharmaceuticals-2810324-supplementary.pdf]

Supplementary data

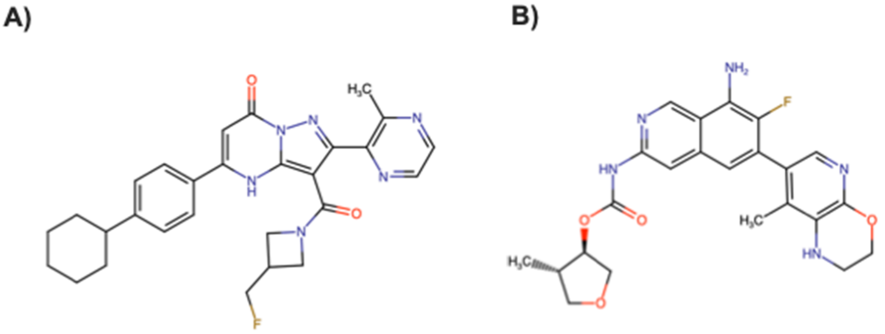

Figure S1: Chemical structures of (a) 7883 and (b) G6893

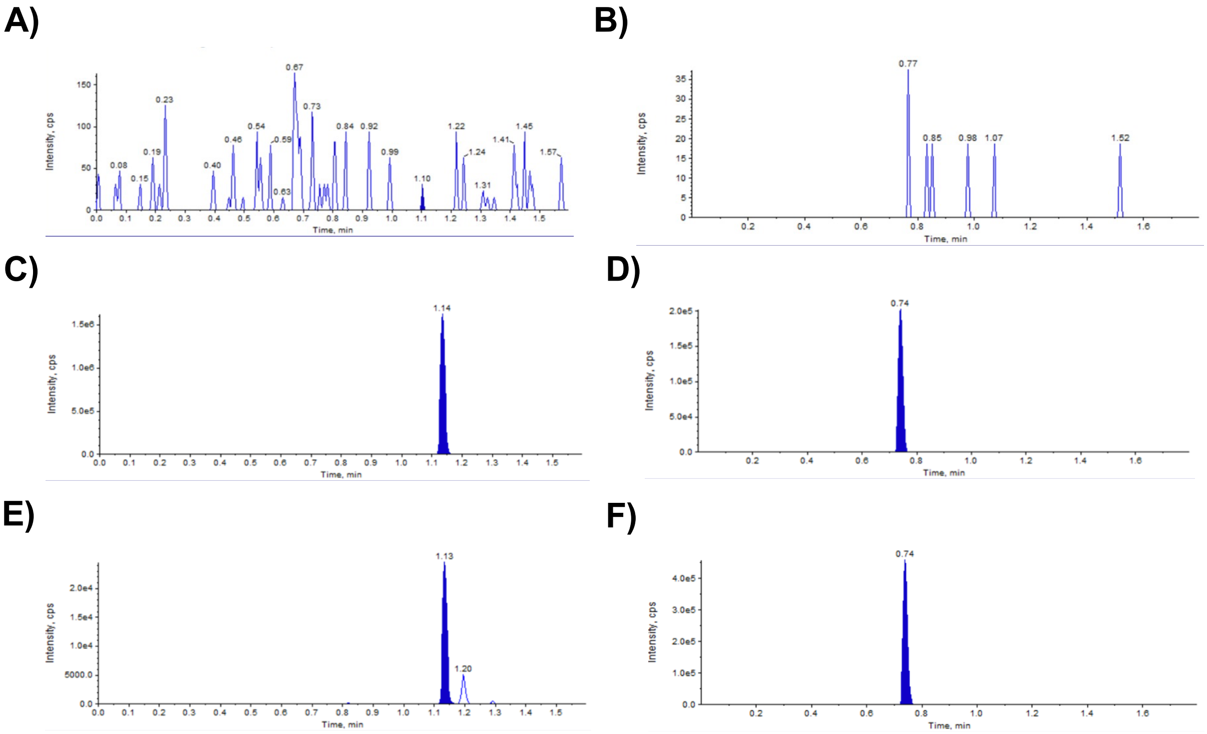

Figure S2: Chromatograms of Blank (a) G7883 and (b) G6893, a standard (c) G7883 and (d) G6893, and a sample (e) G7883 and (f) G6893
